# Activation of GABA_B_ receptors attenuate Alzheimer's disease related pathophysiology in a preclinical APP/PS1 transgenic model

**DOI:** 10.1002/alz70855_099758

**Published:** 2025-12-23

**Authors:** Colin J McArdle, Abigail Caudill, Kimberly F Raab‐Graham

**Affiliations:** ^1^ Wake Forest University School of Medicine, Winston‐Salem, NC, USA

## Abstract

**Background:**

Alongside the major hallmarks of Alzheimer's disease (AD) – amyloid beta (Aβ) plaque, tau tangles, and neurodegeneration – hippocampal synapse loss also presents as a pathological phenotype in this disorder. While there is extensive research linking synapse loss to hyperexcitability and glutamate excitotoxicity, there is a gap in knowledge as to how other neurotransmitters systems, including the inhibitory GABAergic system, are responding to elevated levels of synapse loss and excitatory signaling in AD. Therefore, the goal of this project is to examine the role of metabotropic GABA_B_ receptors (GABA_B_Rs) towards hippocampal synapse loss, as well as additional AD‐related pathologies.

**Methods:**

The APP/PS1 transgenic model were used to investigate the role of GABA_B_Rs in AD. To determine the effect of GABA_B_Rs towards AD‐related pathologies, 4‐6‐month‐old WT and APP/PS1 mice received a single injection of either 1mg/kg baclofen (GABA_B_R agonist), 10mg/kg CGP35348 (GABA_B_R antagonist), or vehicle for one hour, prior to immunostaining or behavior. Forced Swim Test, Splash Test, and Sucrose Preference Test were used to determine the effects of treatment on depressive‐like behaviors.

**Results:**

Utilizing the APP/PS1 model, our results first indicate the presence of deficits in synaptic engagement and a hyperexcitable phenotype, highlighting the validity of this model to investigate these two pathologies. In parallel with these alterations, APP/PS1 models display an increase in total dendritic expression of both principal GABA_B_R subunits, GABA_B1_R and GABA_B2_R, with a contradicting decrease in GABA_B_R surface membrane expression and function. To determine the distinct role of GABA_B_Rs in AD, wildtype and APP/PS1 mice underwent a single treatment with either baclofen, CGP35348, or vehicle. Interestingly, a 1mg/kg dose of baclofen in APP/PS1 mice restored hippocampal synapse number back to wildtype levels, while a 10mg/kg dose of CGP35348 had no effect. Expanding on the results that pharmacological activation of GABA_B_Rs is therapeutic towards synapse loss in AD, our next direction was to determine if GABA_B_Rs could alleviate other AD‐related pathologies. Furthermore, GABA_B_R activation was shown to reduce hippocampal hyperexcitability, mitigate reactive gliosis, and elicit an anti‐depressive behavioral effect in APP/PS1 mice.

**Conclusion:**

Collectively, these results emphasize a neuroprotective role of GABA_B_R‐mediated signaling towards the pathogenesis and symptomology of AD.

